# What if the future of HER2‐positive breast cancer patients was written in miRNAs? An exploratory analysis from NeoALTTO study

**DOI:** 10.1002/cam4.4449

**Published:** 2021-12-17

**Authors:** Sara Pizzamiglio, Giulia Cosentino, Chiara M. Ciniselli, Loris De Cecco, Alessandra Cataldo, Ilaria Plantamura, Tiziana Triulzi, Sarra El‐abed, Yingbo Wang, Mohammed Bajji, Paolo Nuciforo, Jens Huober, Susan L. Ellard, David L. Rimm, Andrea Gombos, Maria Grazia Daidone, Paolo Verderio, Elda Tagliabue, Serena Di Cosimo, Marilena V. Iorio

**Affiliations:** ^1^ Bioinformatics and Biostatistics Unit Department of Applied Research and Technological Development Fondazione IRCCS Istituto Nazionale dei Tumori Milan Italy; ^2^ Molecular Targeting Unit Department of Research Fondazione IRCCS Istituto Nazionale dei Tumori Milan Italy; ^3^ Integrated Biology Platform Department of Applied Research and Technological Development Fondazione IRCCS Istituto Nazionale dei Tumori Milan Italy; ^4^ Breast International Group (BIG) Brussels Belgium; ^5^ Novartis Pharma AG Basel Switzerland; ^6^ Institute Jules Bordet (IJB) Brussels Belgium; ^7^ Molecular Oncology Group Vall d'Hebron Institute of Oncology (VHIO) Barcelona Spain; ^8^ Breast Center Cantonal Hospital St. Gallen Switzerland; ^9^ BC Cancer Kelowna British Columbia Canada; ^10^ Department of Pathology Yale University School of Medicine New Haven Connecticut USA; ^11^ Biomarkers Unit Department of Applied Research and Technological Development Fondazione IRCCS Istituto Nazionale dei Tumori Milan Italy

**Keywords:** biomarkers, breast cancer, HER2, microRNA, trastuzumab

## Abstract

**Background:**

Neoadjuvant therapy with dual HER2 blockade improved pathological complete response (pCR) rate in HER2‐positive breast cancer patients. Nevertheless, it would be desirable to identify patients exquisitely responsive to single agent trastuzumab to minimize or avoid overtreatment. Herein, we evaluated the predictive and prognostic value of basal primary tumor miRNA expression profile within the trastuzumab arm of NeoALTTO study (ClinicalTrials.gov Identifier: NCT00553358).

**Methods:**

RNA samples from baseline biopsies were randomized into training (*n* = 45) and testing (*n* = 47) sets. After normalization, miRNAs associated with Event‐free survival (EFS) and pCR were identified by univariate analysis. Multivariate models were implemented to generate specific signatures which were first confirmed, and then analyzed together with other clinical and pathological variables.

**Results:**

We identified a prognostic signature including *hsa‐miR‐153‐3p* (HR 1.831, 95% CI: 1.34–2.50) and *hsa*‐*miR*‐*219a*‐*5p* (HR 0.629, 95% CI: 0.50–0.78). For two additional miRNAs (*miR‐215‐5p* and *miR‐30c‐2‐3p*), we found a statistically significant interaction term with pCR (p.interaction: 0.017 and 0.038, respectively). Besides, a two‐miRNA signature was predictive of pCR (*hsa‐miR‐31‐3p*, OR 0.70, 95% CI: 0.53–0.92, and *hsa‐miR‐382‐3p*, OR: 1.39, 95% CI: 1.01–1.91). Notably, the performance of this predictive miRNA signature resembled that of the genomic classifiers PAM50 and TRAR, and did not improve when the extended models were fitted.

**Conclusion:**

Analyses of primary tumor tissue miRNAs hold the potential of a parsimonious tool to identify patients with differential clinical outcomes after trastuzumab based neoadjuvant therapy.

## Graphical abstract

Despite the improved pathological complete response rate in HER2‐positive breast cancer patients with neoadjuvant dual HER2 blockade, it would be desirable to identify patients exquisitely responsive to single agent trastuzumab to minimize or avoid overtreatment. Herein, by analyzing baseline tissue microRNA (miRNA) profile in the trastuzumab arm of NeoALTTO study, we identified a two‐miRNA predictive signature and a prognostic signature associated to event‐free survival. Analyses of primary tumor tissue miRNAs hold the potential of a parsimonious tool to identify patients with differential clinical outcomes after trastuzumab based neoadjuvant therapy.

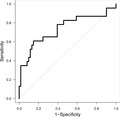

## 1 INTRODUCTION

HER2 amplification and/or overexpression occur in 20% of breast cancer (BC) cases, and it is associated with poor disease outcome. The humanized anti‐HER2 antibody trastuzumab has significantly improved the survival of early HER2+ BC patients, however up to a quarter of cases eventually relapse. Dual blockade with different compounds against HER2, such as lapatinib or pertuzumab^1^, ^2^ has improved the activity and ameliorated disease outcome, however it might be unnecessary in patients who would benefit from single‐agent trastuzumab.

Tumor dependence on HER2^3^ or immune infiltrate^4^ might serve as predictive biomarkers, however elucidating the molecular pathways involved in trastuzumab resistance has been difficult due to the variety of mechanisms of action of this drug. HER2 status in primary tumor still remains the only biomarker used in clinical practice.^5^

MicroRNAs (miRNAs) have been correlated with occurrence and progression of human cancer,^6^ and their potential as clinical tools has recently emerged. However, the approach mainly applied to date to elucidate the mechanisms of resistance to anti‐HER2 therapies has been miRNA modulation in vitro,^7^, ^8^ whereas only few reports have investigated the effects in vivo in mouse models, and even fewer have evaluated miRNA expression in clinical specimens in association with trastuzumab response.^9^, ^10^

In the present study, we extensively profiled primary tumor tissue miRNAs from the NeoALTTO trial^1^ to define miRNA signatures able to identify patients deriving benefit from neoadjuvant single‐agent trastuzumab.

## 2 MATERIALS AND METHODS

### 2.1 Patients

In the multicenter randomized phase III NeoALTTO trial (NCT00553358) HER2+ BC patients were randomized to preoperative lapatinib, trastuzumab, or the combination as previously described.^1^ The primary endpoint of the study was pathological complete response (pCR); the secondary endpoint was event‐free survival (EFS), defined as the time from randomization to first event (BC relapse after surgery, second primary malignancy, death, or failure to complete neoadjuvant therapy because of disease progression).

### 2.2 Sample collection and processing

RNA samples were obtained from snap‐frozen core biopsies of primary tumors before the initiation of neoadjuvant therapy and stored at the central biobank of Vall d'Hebron University Hospital, Barcelona before shipping to INT (Istituto Nazionale dei Tumori of Milan).

Extracted RNA samples underwent quality control assessment determined by measuring the RNA integrity number (RIN) using the RNA tape on a Tapestation 4200 (Agilent) and were quantified with a Qubit Fluorometer (Thermo Fisher). Samples having RIN > 6 were profiled for miRNA expression.

cDNA synthesis and quantitative real‐time PCR were performed according to the manufacturer's protocol. The polyadenylation and reverse transcription were carried out starting from 40 ng of total RNA with the miRCURY LNA cDNA synthesis kit II (Exiqon) in 40 µl reactions. cDNA reactions were mixed 1:1 with ExiLENT SYBR Green master mix (Exiqon). ROX Reference Dye (Invitrogen) was added at 50 nM to the final mixture. Ten microliters of PCR reactions were added to the ready‐to‐use miRNome miRNA panels I and II (Exiqon) spotted into 384‐well plates, which collectively detect 752 human small RNA targets.

Amplification was performed on a QuantStudio 12K Flex Real‐Time PCR System (Applied Biosystems) using the following cycling conditions: 95°C for 10 min followed by 40 amplification cycles at 95°C for 10 s and 60°C for 10 s. Data were acquired using the QuantStudio 12K Flex Software v1.2.2.

**FIGURE 1:**
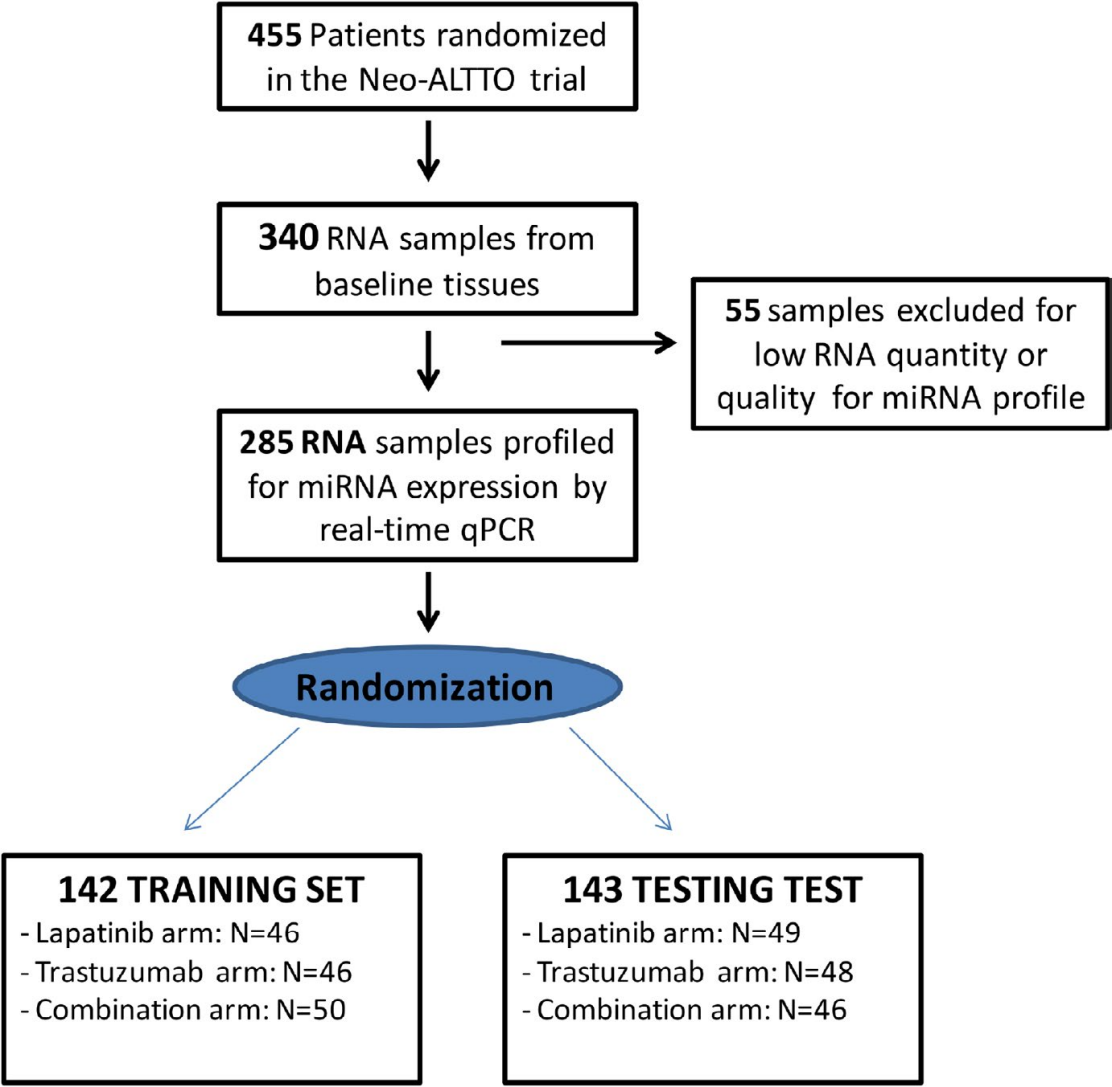
NeoALTTO miRNA analysis flow diagram of frozen tissue samples. Following quality check, 285 out of 340 RNA samples considered suitable for miRNA profile. Samples then randomized in a training set (*n* = 142) and a testing (*n* = 143) set

Out of 340 RNA samples received, a total of 55 were excluded because of low quality, and the remaining 285 splitted in three treatment arms were analyzed for miRNA profiling and randomized in a training set (*n* = 142) and a testing set (*n* = 143) (Figure 1). According to the aim of the present study only patients within the trastuzumab arm were considered. One additional sample of the training set and one sample of the testing set in the trastuzumab arm were further excluded from the statistical analysis because of undetected Ct values of cel‐miR‐39‐3p RNA spike‐in control (quality check failed). Table 1 reports clinico‐pathological features of training (*N* = 45) and testing (*N* = 47) sets in the trastuzumab arm (study population).

**TABLE 1:** Clinico‐pathological features of training and testing sets in the trastuzumab arm (*N* = 92)

	Training (*n* = 45)		Testing (*n* = 47)		*p* Value^a^
	*N*	%	*N*	%	
Age					
<50	19	42	27	57	0.144
≥50	26	58	20	43	
Tumor size					
≤5	32	71	29	62	0.339
>5	13	29	18	38	
Lymphonodal status					
N0	14	31	12	26	0.552
≥N1	31	69	35	75	
ER status					
Negative	23	51	29	62	0.306
Positive	22	49	18	38	
pCR					
Yes	13	29	13	28	0.896
No	34	71	34	72	
Events					
Yes	15	33	12	26	0.411
No	30	67	35	74	

Note

Among the 94 RNA samples from patients treated with trastuzumab (study population) one sample of the training set and one sample of the testing set were excluded from the statistical analysis after quality check of the data.

Abbreviations: ER, estrogen receptor; pCR, pathological complete response.

^a^ Chi‐squared test.

### 2.3 miRNA profiling

miRNA expression has been evaluated using the Exiqon miRCURY LNA™ Universal RT microRNA PCR system and microRNA Ready‐to‐Use PCR, Human panel I+II, which allowed the evaluation of 752 microRNAs. Panel I was designed by the manufacturer and contains high‐priority miR primer sets. These miRNAs are generally highly expressed, highly cited in the literature, and likely to be differentially expressed in disease. Following the same criteria, panel II complements primer sets for other important miRs not included in Panel I.

### 2.4 Statistical analysis

#### 2.4.1 Pre‐processing and univariate analysis

The considered samples were divided into a training set (*n* = 45) and a testing (*n* = 47) set. Within each set of data (i.e., training and testing sets) the relative quantity (RQ) of each miRNA was computed using the comparative threshold cycle method following the formula 2^−ΔCt^, with ΔCt = Ct miRNA[*i*] − Ct reference. For this step, data were normalized according to both the snoRNU38 and the overall mean (OM) approaches.^11^ Training set data were then analyzed in univariate fashion to identify candidate miRNAs, that is, statistically associated with the considered clinical outcome (pCR or events). In this selection step, only those miRNAs detected in at least 10 cases (pCR and events, respectively) were considered in the statistical analysis^12^ according to rule of thumb proposed by Harrell that suggests to implement model with at least 10 events for each variable included in the model.^13^

The relationship between each selected miRNA and the considered clinical outcome was investigated by resorting to regression models based on restricted cubic splines. miRNAs expression normalized (log_2_RQ) on snoRNU38 showing a statistical significance in univariate analysis was considered for signature building in multivariate fashion, if their significance was retained (at alpha level of 10%) even using the OM. According to the required number of event per variable, standard or penalized estimations were used to generate multivariate models (i.e., signatures) by means to an all‐subset analysis.^13^

#### 2.4.2 Prognostic signature

In the training set, the association between miRNA levels and EFS was assessed by resorting to a univariate Cox regression model^14^ in order to select those miRNAs to be considered in multivariate fashion for signature building. For each signature, the C‐statistic (and its 95% CI) computed according to Uno et al.^15^ was used as pivotal measure for evaluation of the model performance. Signatures with a statistically significant performance (i.e., with the lower 95% CI of the C‐statistics >0.50) in the training set were then evaluated on the testing set. Only signatures retaining a statistically significant performance in the testing set were identified as the best signature(s). Subsequently, by considering the whole study population (i.e., training and testing sets together), each of the available clinico‐pathologic variables was singly added to the selected signature(s). In this way for each of the available clinico‐pathologic variable was generated an extended (multivariate) model, the performance of which was then evaluated in terms of C‐statistic. A 30‐week landmark analysis was performed when pCR was considered. Finally, in order to preliminarily identify miRNAs with a different level of expression according to pCR status, on the whole study population we implemented for each miRNA a multivariate Cox regression model including the expression level of the considered miRNA and the pCR status (main effects) together with their first‐order interaction term.

#### 2.4.3 Predictive signatures

In the training set, the association between miRNA levels and pCR was assessed by resorting to a logistic regression model in univariate fashion in order to select those miRNAs to be jointly considered in multivariate fashion for signature building. For each signature, the area under the ROC curve (AUC) and its corresponding 95% confidence interval (95% CI) were calculated. Signatures showing a statistically significant performance (i.e., lower 95% CI of AUC > 0.50) in the training set were then assessed in the testing set. Signatures retaining a statistically significant performance even in the testing set were identified as the *best* signature(s). Finally, by considering the whole study population, we assessed the role of the available clinico‐pathological variables. The performance of the extended models including the miRNA signature and the clinico‐pathologic variables was eventually assessed. Lastly, the predictive performance of the miRNA signature together with that of the molecular classifiers (i.e., TRAR and PAM50) previously reported as driver of PCR was evaluated.

All statistical analyses were carried out with the SAS (version 9.4.; SAS Institute, Inc.) and R software (version 3.6.0; R Foundation for Statistical Computing) by adopting a significance alpha level of 5%.

## 3 RESULTS

### 3.1 Basal miRNA expression profile and EFS

In the training set, 23 out of 608 miRNAs considered for the analysis (i.e., miRNAs detected in at least 10 cases with event) were significantly associated with EFS by univariate analysis (Table 2). By combining these miRNAs into multivariate models following all‐subset analysis approach,^13^ we identified a final signature of two miRNAs with a significant performance in the training set that was confirmed in the testing set and with the best performance in the overall study cohort (*n* = 92).

**TABLE 2:** Results of the univariate Cox regression model‐training set

miRNAs	HR	95% CI
hsa‐miR‐1200	0.671	0.512; 0.879^a^
hsa‐miR‐1238‐3p	0.669	0.543; 0.826^a^
hsa‐miR‐1265	0.839	0.705; 0.997^a^
hsa‐miR‐129‐5p	0.785	0.628; 0.980^a^
hsa‐miR‐153‐3p	1.412	1.026; 1.943
hsa‐miR‐1539	0.826	0.697; 0.979^a^
hsa‐miR‐1908‐5p	0.796	0.648; 0.978^a^
hsa‐miR‐205‐5p	0.737	0.553; 0.982
hsa‐miR‐219a‐5p	0.787	0.627; 0.989
hsa‐miR‐25‐5p	0.566	0.391; 0.819^a^
hsa‐miR‐300	0.581	0.399; 0.845^a^
hsa‐miR‐382‐3p	0.791	0.636; 0.985
hsa‐miR‐492	0.774	0.635; 0.944
hsa‐miR‐519a‐3p	0.834	0.696; 0.998^a^
hsa‐miR‐551b‐5p	0.666	0.452; 0.981^a^
hsa‐miR‐583	0.727	0.549; 0.963^a^
hsa‐miR‐600	1.373	1.03; 1.829
hsa‐miR‐626	0.819	0.673; 0.998^a^
hsa‐miR‐651‐5p	0.611	0.382; 0.977
hsa‐miR‐761	0.845	0.721; 0.991
hsa‐miR‐891b	0.658	0.479; 0.904^a^
hsa‐miR‐92a‐2‐5p	0.787	0.637; 0.971^a^
hsa‐miR‐937‐3p	0.748	0.578; 0.967^a^

Abbreviations: CI, confidence interval; HR, hazard ratio.

^a^ microRNAs retained its statistical significance (at 10%) also when normalized for the overall mean.

This signature included miR‐153‐3p (HR 1.83, 95% CI: 1.34–2.50) and miR‐219a‐5p (HR 0.629, 95% CI: 0.51–0.79) leading to a C‐statistics of 0.730 (95% CI: 0.63–0.83). Notably, these two miRNAs retained their statistical significance with respect to EFS even after adjusting for clinico‐pathological variables (Table 3). No statistically significant relationships were observed between the expression level of the two miRNAs and the available clinico‐pathological variables (Table S1, upper part).

**TABLE 3:** Multivariate Cox regression analyses with clinico‐pathological variables

Model	HR	95% CI	C‐statistic	95% CI
hsa‐miR‐153‐3p	1.856	1.352; 2.565	0.721	0.623; 0.820
hsa‐miR‐219a‐5p	0.622	0.501; 0.794		
ER status (Neg vs. Pos)	0.830	0.367; 1.894		
hsa‐miR‐153‐3p	1.932	1.377; 2.799	0.737	0.637; 0.837
hsa‐miR‐219a‐5p	0.619	0.501; 0.788		
Lymph nodal status (≥N1 vs. N0)	1.429	0.601; 3.738		
hsa‐miR‐153‐3p	1.851	1.352; 2.552	0.724	0.623; 0.825
hsa‐miR‐219a‐5p	0.619	0.499; 0.790		
Tumor size (>5 vs. ≤5)	1.444	0.645; 3.175		
hsa‐miR‐153‐3p	1.907	1.373; 2.706	0.733	0.635; 0.832
hsa‐miR‐219a‐5p	0.609	0.484; 0.784		
Age (≥ 50 vs. <50)	0.659	0.275; 1.483		
hsa‐miR‐153‐3p^a^	1.938	1.387; 2.763	0.732	0.632; 0.833
hsa‐miR‐219a‐5p^a^	0.618	0.499; 0.785		
pCR (Yes vs. No)^a^	0.549	0.185; 1.363		

Abbreviations: CI, confidence interval; ER, estrogen receptor; pCR, pathological complete response.

^a^ Landmark analysis on 77 patients and 25 events was performed.

We next identified miRNAs potentially associated with EFS according to pCR status, which is the main driver of EFS in the NeoALTTO and other neoadjuvant studies^16^ in the overall study cohort. For each of the 608 miRNAs, a multivariate model with the miRNAs expression, pCR status, and the first‐order interaction between miRNA and pCR was implemented.

We found two miRNAs (miR‐215‐5p and miR‐30c‐2‐3p) with a statistically significant interaction term with pCR (p.interaction: 0.017 and 0.038, respectively, Figure S1).

For merely exploratory purposes, we pursued our analysis by adding each of the two interaction terms together with the corresponding main effects to the two‐miRNAs signature. As reported in Table S2, the highest prognostic performance in terms of C‐statistics was observed for the multivariate model with *hsa‐miR‐153‐3p*, *hsa‐miR‐219a‐5p*, *miR‐30c‐2‐3p*, pCR, and the first‐order interaction term (miR‐30c‐2‐3p*pCR).

### 3.2 Baseline miRNA expression profile and pCR

**FIGURE 2:**
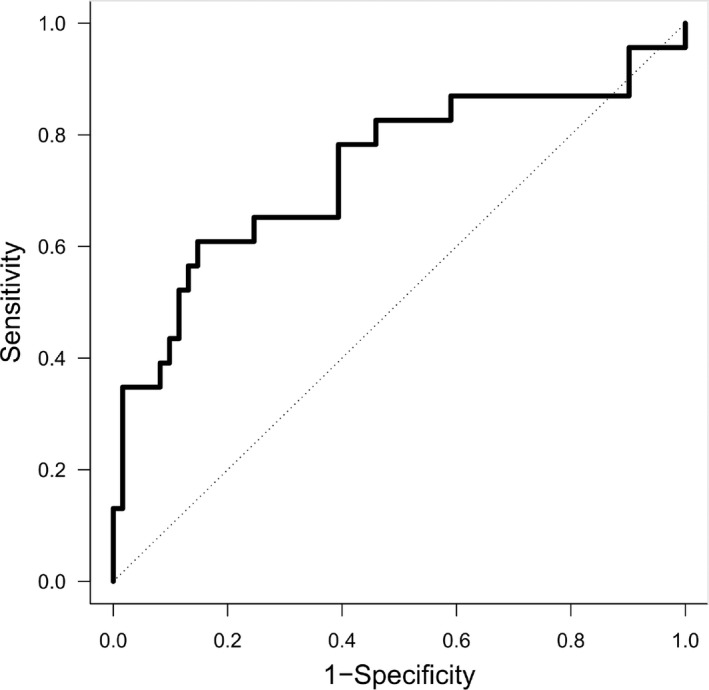
Receiver operating characteristic (ROC) curve of the final signature of miR‐31‐3p and miR‐382‐3p

From the univariate analysis, eight out of 502 considered miRNAs were statistically significantly associated with pCR in the training set. Table 4 shows the OR and 95% CI of the eight identified miRNAs. By combining these miRNAs into multivariate models following all‐subset analysis approach,^13^ we identified a final signature of two miRNAs (*hsa‐miR‐31‐3p*, OR 0.70, 95% CI: 0.53–0.92 and *hsa‐miR‐382‐3p*, OR 1.39, 95% CI: 1.01–1.91) with a significant performance in the training set that was confirmed in the testing set and with the best performance in the overall study with an AUC value of 0.73 (95% CI: 0.60–0.87) (Figure 2). These two miRNAs retained their statistical significance with respect to pCR even after adjusting for other clinico‐pathological variables (Table 5). No statistically significant relationships were observed between the expression level of the two miRNAs and the available clinico‐pathological variables (Table S1, lower part).

**TABLE 4:** Results of the univariate logistic model‐training set

miRNAs	OR	95% CI
hsa‐miR‐132‐3p	0.410	0.169; 0.991
hsa‐miR‐23b‐5p	0.520	0.316; 0.856
hsa‐miR‐31‐3p	0.566	0.351; 0.912
hsa‐miR‐31‐5p	0.508	0.291; 0.887
hsa‐miR‐330‐3p	0.501	0.251; 0.999
hsa‐miR‐34b‐3p	0.673	0.474; 0.956
hsa‐miR‐382‐3p	1.392	1.006; 1.925^a^
hsa‐miR‐548j‐5p	1.702	1.090; 2.658^a^

Abbreviations: CI, confidence interval; OR, odds ratio.

^a^ MicroRNAs retained its statistical significance (at 10%) also when normalized for the overall mean.

**TABLE 5:** Multivariate logistic regression analyses with clinico‐pathological variables

Model	OR	95% CI	AUC	95% CI
hsa‐miR‐31‐3p	0.734	0.559; 0.963	0.769	0.653; 0.885
hsa‐miR‐382‐3p	1.329	0.967; 1.826		
ER status (Neg vs. Pos)	3.147	1.022; 9.689		
hsa‐miR‐31‐3p	0.71	0.541; 0.932	0.746	0.618; 0.875
hsa‐miR‐382‐3p	1.369	1.005; 1.867		
Lymph nodal status (≥N1 vs. N0)	0.476	0.16; 1.414		
hsa‐miR‐31‐3p	0.729	0.556; 0.957	0.739	0.608; 0.871
hsa‐miR‐382‐3p	1.377	1.009; 1.878		
Tumor size (>5 vs. ≤5)	1.543	0.545; 4.375		
hsa‐miR‐31‐3p	0.717	0.543; 0.945	0.743	0.606; 0.879
hsa‐miR‐382‐3p	1.375	1.001; 1.889		
Age (≥50 vs. <50)	0.855	0.300; 2.433		

Abbreviations: CI, confidence interval; ER, estrogen receptor; OR, odds ratio.

### 3.3 Predictive miRNA signature and molecular classifiers

The added value of TRAR and PAM50, which independently predict pCR,^17^ was finally investigated in a subset of samples with both miRNA and molecular classifier data available (*n* = 64). No significant relationship was observed between the two miRNAs and the molecular classifiers. Interestingly, miRNAs appeared to resemble the predictive performance of the TRAR classifier and even to overcome that of PAM50. Furthermore, the ROC curves of extended models including either TRAR or PAM50 showed no significant increment in the predictive capability of the two miRNA signatures (Figure S2).

## 4 DISCUSSION

By analyzing miRNA expression profile in baseline primary tumor tissue, we found miR‐153‐3p and miR‐219a‐5p significantly associated with EFS, and miR‐215‐5p and miR‐30c‐2‐3p interacting with pCR for EFS leading to a C‐statistics ~0.80 when these terms were considered in the final models.

Moreover, we identified two miRNAs, miR‐31‐3p and miR‐382‐3p, significantly associated with pCR even after adjusting for clinico‐pathological features. To date, none of these miRNAs have been related to mechanism of resistance to trastuzumab. However, miR‐31 has been associated with resistance to EGFR‐targeted therapies,^18^ whereas miR‐382 is a known oncosuppressor in pancreatic and ovarian cancers.^19^ Notably, despite these results are preliminary, the discriminatory capability of these two miRNAs is such that they could be a viable and thrifty alternative to well established predictive genomic classifiers, including TRAR and PAM50. Indeed, even though in a small subset of samples for which both miRNA and gene expression were available, miRNAs appeared to resemble the predictive performance of the TRAR classifier and even to overcome that of PAM50.

Both predictive and prognostic signatures were not significant in the other two NeoALTTO study arms (Figure S3), underlining that the performance of these miRNAs as biomarkers might be specific for trastuzumab treatment, and suggesting that their potential functional role might be related to the mechanisms of action of this drug.

We recognize that this study has some limitations, as the sample size, lower than expected due to samples availability as well as to RNA quantity/quality issues. Moreover, we decided to focus only on the trastuzumab arm to perform our miRNA signature discovery. However, even though these results are preliminary and need to be confirmed in independent case series, we are convinced that the clinical outcome of patients treated with neoadjuvant trastuzumab might be better defined by adding miRNA analysis to the information derived from pCR. Furthermore, tissue miRNA analysis could possibly assist in the decision of postsurgical treatment after neoadjuvant trastuzumab, since lack of pCR (and pCR) is unlikely to recapitulate patient prognosis at the individual level. We might thus speculate that miRNAs could be useful to better define patient residual risk after surgery according to the efficacy of neoadjuvant treatment.

In conclusion, this study defined two primary tissue miRNA signatures holding the potential of a parsimonious tool able to predict the benefit of trastuzumab‐based neoadjuvant therapy, and confirms the need to use new markers to better define the outcome of patients treated with neoadjuvant therapy regardless of their response to surgery.

## CONFLICT OF INTEREST

Dr. S. Di Cosimo received speaking fees from Novartis Pharma, and Pierre‐Fabre outside this work and is the recipient of the IG 20774 from Fondazione Associazione Italiana Ricerca sul Cancro (AIRC). Dr. S. El‐abed received grant from Novartis during the conduct of the study, and grants from Roche/Genentech and Pfizer outside the submitted work. Dr. Nuciforo received grants from Novartis for the submitted work and from Novartis, Roche/Genentech, MSD Oncology, Bayer, and Targos outside the submitted work. Dr. Rimm has no conflict of interest related to this work. Unrelated to the topic of the paper, Dr. Rimm has received honoraria and/or grants and/or instrument support from Akoya, Amgen, AstraZeneca, BMS, Cell Signaling Technology, Cepheid, Danaher, Konica/Minolta, Lilly, Merck, NanoString, Navigate Biopharma, NextCure, Odonate, Paige.AI, Roche, Sanofi, and Ventana. None of that funding was related to this work. Dr. Gombos: no conflict of interest related to this publication. Other COI: advisory board (Institution): Lilly, Daiichi Sankyo; travel grants: Pfizer. Dr. Huober J received research funding from Celgene, Novartis, Hexal, Lilly; honoraria from Lilly, Novartis, Roche, Pfizer, AstraZeneca, MSD, Celgene; Eisai, Abbvie, Seagen, Gilead; has consulting advisory relationship with Lilly, Novartis, Roche, Pfizer, Hexal, AstraZeneca, MSD, Celgene, Abbvie, Seagen, Gilead; travel expenses from Roche, Pfizer, Novartis, Celgene, Daiichi. Dr. Wang Y is employee at Novartis and holds Novartis stock. Dr. M Bajji's institution, Institut Jules Bordet, has received a research grant for the conduct of the NeoALTTO study. The other authors have no conflict of interest to declare.

## AUTHOR CONTRIBUTION

Sara Pizzamiglio performed the analyses and wrote the original draft; Giulia Cosentino evaluated the results and wrote the original draft; Chiara M. Ciniselli performed the analyses and edited the manuscript: Loris De Cecco performed the miRNA profile; Alessandra Cataldo, Ilaria Plantamura, and Tiziana Triulzi contributed to data discussion and manuscript editing; Sarra El‐abed, Yingbo Wang, Mohammed Bajji, Paolo Nuciforo, Jens Huober, Susan L. Ellard, David L. Rimm, and Andrea Gombos edited the manuscript; Maria G. Daidone and Elda Tagliabue discussed the data and reviewed the manuscript; Paolo Verderio performed the analyses, discussed the data, wrote the original draft, and edited the manuscript; Serena Di Cosimo and Marilena V Iorio conceived the work, evaluated and discussed the data, wrote the original draft, and edited the manuscript.

## ETHICAL APPROVAL STATEMENT

The study was approved by the TransALTTO committee and was conducted in accordance with the Declaration of Helsinki. The trial was approved by relevant ethics committees and health authorities at all participating sites. Informed consent, including the participation to future biomarker research, was obtained from all participants.

## INFORMED CONSENT

Patients' participation in this sub‐study was allowed after signing the main study consent form, which included a non‐specific clause for use of tissue samples for biomarker research.

## Supporting information

Supplementary Material

## Data Availability

The data that support the findings of this study are available from the corresponding author upon request.
