# Supplementary material for: What if the future of HER2‐positive breast cancer patients was written in miRNAs? An exploratory analysis from NeoALTTO study
Source: Cancer Med. 2021 Dec 17;11(2):332–9. doi: 10.1002/cam4.4449 (PMC8729061; doi:10.1002/cam4.4449)
Supplement: Supplementary file 1 — Supplementary Material [file CAM4-11-332-s001.docx]

**Table S1.** **Association of individual clinic-pathological features** and the expression of the two miRNAs included in the signature associated with event free survival (upper part) and the other two miRNAs included in the signature predicting pCR (lower part).

|  | p.value KW test | |
| --- | --- | --- |
| Clin var | miR-153-3p | miR-219a-5p |
| ER status | 0.69 | 0.62 |
| Lymphonodal status | 0.28 | 0.76 |
| Tumor size | 0.47 | 0.82 |
| Age | 0.33 | 0.28 |
| pCR | 0.20 | 0.98 |
|  |  |  |
| Clin var | miR-31-3p | miR-382-3p |
| ER status | 0.49 | 0.39 |
| Lymphonodal status | 0.55 | 0.88 |
| Tumor size | 0.47 | 0.19 |
| Age | 0.24 | 0.24 |

**Table S2**. **Multivariate model performance.**

|  | **C-Statistic** | **95%CI** | |
| --- | --- | --- | --- |
| hsa-miR-153-3p hsa-miR-219a-5p, miR-215-5p, pCR *,* miR-215-5p*pCR | 0.746 | 0.645 | 0.847 |
| hsa-miR-153-3p hsa-miR-219a-5p, miR-30c-2-3p, pCR*,* miR-30c-2-3p*pCR | **0.787** | **0.708** | **0.865** |

*Lendmark analysis on 77 patients and 25 events was performed. Abbreviations: pCR: pathological Complete Response; CI: Confidence Interval

**Figure S1. Estimate Hazard Ratio and 95% Confidence Interval in patients with and without pCR for miR 30c-2-3p and miR 215-5p.**

Red diamond and line indicate Hazard Ratio (HR ) and its 95% confidence interval (CI) in patients without pathological Complete Response (pCR); blue diamond and line indicate HR and its 95% CI in patients with pCR.


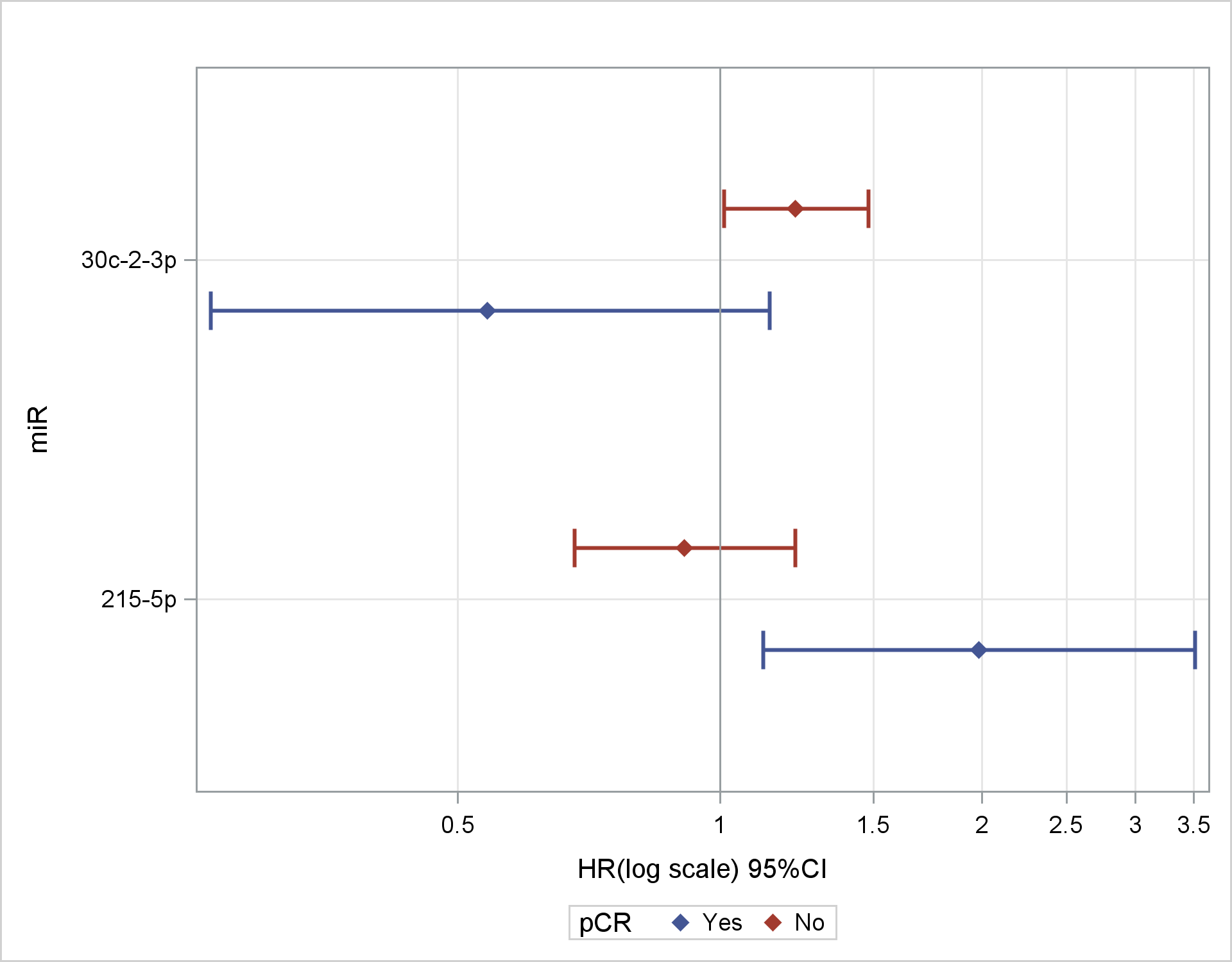


**Figure S2. Predictive performance of the miRNA-signature, TRAR, PAM50 and extended models including TRAR or PAM50.**

Predictive performance in terms of area under the receiver operating characteristic curve (AUC) of the model with the miRNA-signature, TRAR, PAM50 and the extended models including the miRNA-signature, TRAR or PAM50. AUC values are computed on the subset of samples with both miRNA and molecular classifier data available (n=65). For each model dots represent AUC values and the horizontal lines represent the corresponding 95% confidence interval (CI). #definition of the AUC for binary predictors.

**Figure S3. Performance of the two-identified signature in the other arms of NeoALTTO study (Lapatinib and Combination of Trastuzumab+Lapatinib).** Black dot and line indicate C-index (Panel A) or AUC (panel B) with the corresponding 95% confidence interval (CI) in patients randomized in the **other arms of NeoALTTO study** .
